# The immune battlefield: The impact of inflammatory cytokines on CD8^+^ T-cell immunity

**DOI:** 10.1371/journal.ppat.1006618

**Published:** 2017-10-26

**Authors:** Stephanie A. Condotta, Martin J. Richer

**Affiliations:** Department of Microbiology and Immunology, Microbiome and Disease Tolerance Centre, Rosalind and Morris Goodman Cancer Research Centre McGill University, Montreal, Quebec, Canada; Duke University School of Medicine, UNITED STATES

## Soldiers of the immune system: CD8^+^ T-cell basics

**Fig 1:**
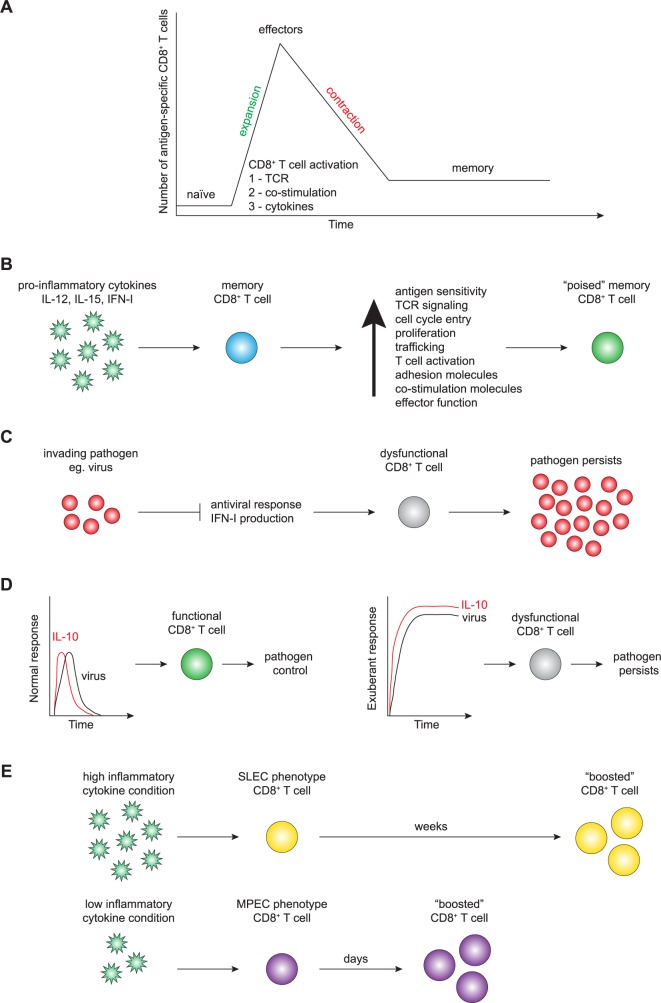
**A.** CD8^+^ T-cell basics: upon integrating the 3 signals needed for activation (1, TCR; 2, costimulation; 3, cytokines), a naïve antigen-specific CD8^+^ T cell clonally expands, acquires effector functions to kill invading pathogen, and then undergoes numerical contraction, developing into long-lived protective memory antigen-specific CD8^+^ T cells. **B**. Memory CD8^+^ T-cell exposure to proinflammatory cytokines (i.e., IL-12, IL-15, IFN-I) enhances their function, allowing them to counteract and neutralize pathogen risk. **C.** Virus-mediated inhibition of IFN-I production leads to dysfunctional CD8^+^ T-cell responses and pathogen persistence. **D**. Transient IL-10 does not impair functional CD8^+^ T-cell responses, allowing pathogen control and clearance. Sustained IL-10 production leads to CD8^+^ T-cell dysfunction and pathogen persistence. **E**. Inflammation influences memory CD8^+^ T-cell generation, impacting vaccine development and protective immunity. IFN, interferon; IL, interleukin; MPEC, memory precursor effector cell; SLEC, short-lived effector cell; TCR, T-cell receptor.

Within the microscopic world, there is an ongoing war between the host and foreign invaders, and the battlefield is the immune system. When a pathogen encounters innate immune cells, such as dendritic cells (DCs), it is quickly recognized as foreign. The DCs provide alarm signals in the form of inflammatory cytokines, degrade the pathogen, and present pathogen-derived peptides to adaptive immune cells, including CD8^+^ T cells. The primary function of CD8^+^ (or cytotoxic) T cells is to recognize and kill infected cells. They are critical soldiers of the immune system, but before carrying out their function, they must receive 3 signals to become appropriately activated. A naïve CD8^+^ T cell must have T-cell receptor (TCR) engagement by peptide:major histocompatibility complex (MHC) complexes from the antigen-presenting cell (signal 1) [1–5], ligation of costimulatory receptors (signal 2) [4, 6–11], and receive specific cytokine signals (signal 3), which are required for optimal cell accumulation, proliferation, and function [9, 12–15]. After integrating the proper signals, the newly activated antigen-specific CD8^+^ T cells undergo clonal expansion [16–18] and develop into “effectors” that have acquired the capacity to kill pathogen-infected host cells [1, 12, 19–22]. Following clonal expansion, 90%–95% of the pathogen-specific effector CD8^+^ T-cell population numerically contracts via apoptosis [18, 23, 24], and the remaining 5%–10% of cells constitute a long-lived memory CD8^+^ T-cell pool that provides enhanced protection from secondary infection (Fig 1A) [18, 23, 25, 26]. The development of the long-lived memory CD8^+^ T-cell pool is not a stochastic event, and the cells that seed this population can be identified among effector T cells early following infection. Thus, effector CD8^+^ T cells can be broadly divided into the following 2 subsets: memory precursor effector cells (MPECs), which will predominantly become long-lived memory CD8^+^ T cells and short-lived effector cells (SLECs), which are mostly lost during contraction after the T-cell response [27]. The establishment of a pool of memory CD8^+^ T cells is the goal of T-cell vaccination strategies, and understanding how to modulate their function is critical for vaccine development and immunotherapies.

## CD8^+^ T-cell battalion: How the host benefits from pathogen-induced inflammation

Following infection, inflammatory cytokines (i.e., signal 3) are induced to counter the invading pathogen and to alarm the immune system. Pathogen-induced inflammatory cytokines impact several aspects of memory CD8^+^ T-cell biology. In addition to serving as signal 3 during the activation of naïve T cells, inflammatory cytokines regulate the antigen sensitivity, proliferation, and trafficking of both effector and established memory T-cell populations, giving the host an advantage over the invading pathogen (Fig 1B) [15, 28–30]. Thus, the capacity of the host to respond to infection by producing inflammatory cytokines is critical for host protection.

Pathogens such as *Listeria monocytogenes* (LM, a gram-positive bacterium) and lymphocytic choriomeningitis virus Armstrong strain (LCMV-Arm; genus *Mammarenavirus*, family Arenaviridae) have been used extensively to determine the requirements for protective CD8^+^ T-cell responses. These model pathogens induce distinct inflammatory cytokine milieu upon infection and have been useful in dissecting the impact of inflammatory cytokines on effector and memory T-cell responses. Following LM infection, several inflammatory cytokines are produced, including interleukin-12 (IL-12), whereas LCMV-Arm elicits primarily a type 1 interferon (IFN-I) response [28]. Inflammatory cytokines triggered by infection (both IL-12 and IFN-I) enhance the capacity of effector and memory CD8^+^ T cells to respond to low concentrations of antigen (antigen sensitivity) [28]. Inflammatory cytokine signaling in CD8^+^ T cells directly enhances TCR signaling capacity and reduces the threshold of antigen required for T-cell activation. Thus, extrinsic inflammatory signals fine-tune the CD8^+^ T-cell response [28]. Additionally, even in the absence of specific antigen, virus-driven IFN-I induces IL-15, which stimulates the entry of memory CD8^+^ T cells into the cell cycle. IL-15 can therefore enhance the proliferative capacity of memory T cells that may otherwise require a higher threshold for their activation [30, 31]. Thus, inflammatory cytokine production by the host is required for an optimal CD8^+^ T-cell response to infection.

Importantly, inflammatory IL-15 also regulates the capacity of memory CD8^+^ T cells to traffic to inflamed tissues in an antigen-independent manner [29]. Nolz and Harty demonstrated that in memory CD8^+^ T cells, *Gcnt1* expression, an enzyme used for generating core 2 O-glycans, is induced by inflammatory IL-15, modulating the capacity of memory CD8^+^ T cells to bind to P- and E-selectin. Epigenetic changes at the *Gcnt1* locus underlie the differential capacity of memory CD8^+^ T cells to traffic in response to inflammatory cytokines as compared with their naïve counterparts [29]. Furthermore, cognate antigen recognition is not strictly required for some memory CD8^+^ T-cell effector functions [32]. For example, when memory CD8^+^ T cells are exposed to inflammatory cytokines without cognate antigen, they become activated and up-regulate trafficking, adhesion, and costimulatory molecules and rapidly exert effector functions (IFNγ, granzyme B, and perforin). Strikingly, these processes are dependent on the induction of IL-15 (Fig 1B) [32]. Together, these results demonstrate the critical role of inflammatory IL-15 in shaping memory CD8^+^ T-cell responses to pathogenic challenge. These data highlight the importance of the interplay between the pathogen and the host inflammatory response that can each dictate the protective capacity of the immune response. The ability of preexisting memory CD8^+^ T cells to modulate their function in response to inflammatory cytokines and independently of cognate antigen suggests that this may serve as an early warning signal to prepare these cells to attack the invading pathogen. This likely represents an important immunomodulatory mechanism that reduces the potential for the induction of immunopathology by memory CD8^+^ T cells at steady state while still allowing for enhanced responsiveness following infection. The presence of this regulatory loop dictated by extrinsic factors leads to an important question regarding whether some pathogens have developed mechanisms to target these same regulatory pathways to dampen immune responses.

## Enemy siege: How the pathogen counters host defenses

The antiviral immune response often begins with the detection of viral RNA by retinoic acid-inducible gene-I (RIG-I) family receptors (RLRs) such as RIG-I and melanoma differentiation-associated protein 5 (MDA5). This initiates mitochondrial antiviral signaling (MAVS), the activation of transcription factors (i.e., IFN regulatory factor [IRF]-3 and IRF-7), and the induction of IFN-stimulating genes (ISGs), leading to the production of IFN-I (i.e., IFNα/β). Because proinflammatory cytokines play a key role in the initiation and regulation of the antiviral immune response, several pathogens have developed strategies to block this critical step. One such strategy is to directly block the production of IFN-I (Fig 1C). For example, several viral proteins (including nonstructural protein 1 [NS1], NS2A, and envelope protein) from West Nile virus (genus *Flavivirus*, family Flaviviridae) can target and inhibit IFN-I induction [33]. Other viruses have also evolved strategies to counteract host defenses; for example, all viruses from the Paramyxoviridae family encode a V protein, which directly binds to MDA5, inhibiting double-stranded RNA (dsRNA) binding and downstream signaling, and the NS1 protein of influenza virus (genus *Influenzavirus A*, family Orthomyxoviridae) prevents optimal downstream signaling of RIG-I [34]. The NS3 viral protease of hepatitis C virus (HCV; genus *Hepacivirus*, family Flaviviridae) cleaves MAVS, abrogating RLR-mediating signaling, and the X protein of hepatitis B virus (HBV; genus *Orthohepadnavirus*, family Hepadnaviridae) targets MAVS for ubiquitination and proteasomal degradation [34]. This inhibition not only affects the capacity of the host to establish an antiviral state but will also impact the quality of the ensuing CD8^+^ T-cell response, which is dependent on signal 3 cytokines such as IFN-I, as described above.

Conversely, some pathogens successfully establish infection by inducing an overexuberant or inappropriate immune response that becomes detrimental for host protection. For example, sustained IFN-I induced during chronic infection (e.g., LCMV Clone 13) leads to sustained IL-10 production, which negatively impacts the developing adaptive immune response (Fig 1D) [35]. The critical role of IL-10 in facilitating viral persistence was shown by the genetic deletion or antibody-mediated blockade of IL-10, both of which restore the capacity of the host to mount an effective antiviral response [36]. The induction of IL-10 has also been linked to viral persistence of other important human pathogens such as HCV [37]. Notably, viruses have also evolved mechanisms to directly evade immunity by encoding anti-inflammatory cytokine homologs that promote latent infections. Herpesviruses (family Herpesviridae), such as human cytomegalovirus (HCMV) and Epstein-Barr virus (EBV), encode IL-10 homologs that dampen the immune response, thereby playing a key role in establishing viral latency. Virus-encoded IL-10 also inhibits proinflammatory cytokine production, which alters cytokine responses and inhibits lymphocyte function to further facilitate virus dissemination and latency [38]. Thus, pathogens that establish chronic infections have developed evasion techniques to promote pathogen survival by activating host immunoregulatory loops and dampening host defenses.

Chronic infections have also been shown to alter immunity to unrelated pathogens and impair vaccine-induced immune responses [39]. As noted, long-lived memory CD8^+^ T cells are enriched among the MPEC subset [40], and chronic bystander infections can hamper the development of memory CD8^+^ T cells [41]. Memory CD8^+^ T-cell populations generated in the presence of chronic inflammation are decreased in numbers, exhibit reduced proportions of MPECs, express lower levels of surface molecules associated with memory formation, and exhibit impaired cytokine production [41]. Chronic infections also negatively impact secondary expansion and protective capacity of developing memory CD8^+^ T cells. These results demonstrate that chronic bystander inflammatory cytokines can exert adverse effects on the memory CD8^+^ T-cell formation. These data also support that pathogens that establish chronic infection can impair T-cell function by manipulating the host’s inflammatory cytokine milieu. These regulatory networks have important implications for understanding both memory CD8^+^ T-cell development and the design of vaccines, especially in host populations where chronic infections are prevalent.

## Immune fortifications: The effects of inflammatory cytokines on vaccine development

Understanding how to modulate the protective characteristics of memory CD8^+^ T cells is of great importance for vaccine development and T-cell–mediated immunotherapies [42, 43]. Because inflammatory cytokines play such a central role in T-cell biology, it is important to consider how these impact vaccination strategies. Vaccine strategies typically require a “boost” following the initial primary antigen exposure in order to generate sufficient memory T-cell numbers to provide protection. However, the logistics of prime-boost strategies require a relatively long interval between immunizations and the induction of an inflammatory response initiated by adjuvants. Conversely, reduced levels of inflammatory signal 3 cytokines can shorten the interval between immunization and booster [44, 45]. As an example, when naïve CD8^+^ T-cell populations are primed in mice either infected with LM (high inflammatory cytokine condition—primarily SLEC population generated) or primed by vaccination with DCs coated with LM peptides (low inflammatory cytokine condition—primarily MPEC population generated), effective boosting can be achieved much earlier in the DC-vaccinated group compared with the LM-infected group (Fig 1E) [44]. These results demonstrate that inflammatory cytokines influence the rate at which CD8^+^ T cells acquire functional characteristics of memory lymphocytes, including their responsiveness to booster immunizations [44].

Although the goal of prime-boost vaccination is to increase numbers of memory CD8^+^ T cells, repeated antigen stimulation can also have a negative impact on memory CD8^+^ T-cell function. For example, with every antigen encounter, the magnitude of the memory CD8^+^ T-cell response is reduced, contraction is protracted, and the basal proliferative capacity of memory CD8^+^ T cells is decreased [46]. Furthermore, repeated antigen stimulation results in reduced expression of multiple cytokine receptors, indicating a change in cytokine responsiveness with each antigen encounter [46]. These results indicate that although prime-boost vaccination can generate a large number of memory CD8^+^ T cells, they may not function properly when challenged with an ensuing pathogen because of their lack of sensitivity to inflammatory cytokines. Recent studies have also demonstrated that CD8^+^ T-cell responses induced in response to subunit vaccination differ from CD8^+^ T-cell responses to infection. Specifically, it was demonstrated that CD8^+^ T cells responding to subunit vaccination are dependent on IL-27 rather than the classical signal 3 cytokines IL-12 and IFN-I [47]. These data further support the notion that our current prime-boost vaccine strategies need to be optimized in order to generate optimal memory CD8^+^ T cells and that we need to gain a better understanding of the impact of the inflammatory milieu during vaccination.

Tissue-resident memory T (Trm) cells, another subset of memory T cells, have been recently identified as playing an important role in protective immunity. As opposed to central memory T cells, these cells do not recirculate but rather reside in specific tissues to provide protection at pathogen entry sites. They can produce IFNγ upon activation, serving as an alarm system and recruiting other immune cells into the infected tissue [48, 49]. These cells are of interest for vaccine development because they are the frontline defense against secondary challenge in tissue-specific areas. For instance, a vaccine strategy targeting Trm cells to mucosal areas, such as the lung or genital tract, could aid in the control of viruses that target these specific tissues [48, 49]. However, the full potential of these cells is not known, and future studies are warranted to further our understanding of the correlates of protection.

## Conclusion

In order to improve immune-mediated control of infection or optimize vaccination, it is critical to understand both the host’s response to pathogens and the evasion tactics pathogens employ to combat these responses. There is mounting evidence showing that pathogen-induced inflammation modulates both the induction of effector CD8^+^ T-cell populations and the protective functions of established memory CD8^+^ T-cell populations. Over time, there has been a constant battle between pathogens and the host, and both have evolved countermeasures to promote their own survival. Understanding the impact of these host–pathogen interactions will lead to new mechanistic approaches to stimulate effective protection from infection.
